# Animals Experimentally Infected with SARS-CoV-2 Generate Functional Autoantibodies against G-Protein-Coupled Receptors

**DOI:** 10.3390/biomedicines11102668

**Published:** 2023-09-28

**Authors:** Gerd Wallukat, Kerstin Wernike, Dipthi Bachamanda Somesh, Thomas C. Mettenleiter, Johannes Müller

**Affiliations:** 1 Berlin Cures GmbH, 13125 Berlin, Germany; jmueller@berlinheals.de; 2 Friedrich-Loeffler-Institut, 17493 Greifswald-Insel Riems, Germany; 3 Berlin Heals, 10719 Berlin, Germany

**Keywords:** viral infection, SARS-CoV-2, long-COVID, autoantibodies, G-protein-coupled receptors, immune response

## Abstract

(1) Background: SARS-CoV-2 infection has been linked to diverse clinical manifestations in humans, including cardiovascular complications. Functional autoantibodies targeting G-protein-coupled receptors have emerged as potential contributors to these effects. This study sought to investigate the production and activity of functional autoantibodies targeting G-protein-coupled receptors after SARS-CoV-2 infection of selected animal species. (2) Methods: The presence of functional autoantibodies such as 2-adrenoceptor, angiotensin II AT1 receptor, muscarinic M2 receptor, and angiotensin 1–7 MAS receptor was assessed in cattle and ferrets experimentally infected with SARS-CoV-2. Bioassays were conducted to evaluate the positive or negative chronotropic responses induced by these autoantibodies. Further experiments identified the extracellular domains to which the functional autoantibodies bind, and receptor antagonists were employed to block the induced responses. (3) Results: Only two out of six cattle that were inoculated with SARS-CoV-2 displayed viral replication and tested positive for functional autoantibodies against G-protein-coupled receptors. These functional autoantibodies specifically recognized β2-adrenoceptor, angiotensin II AT1 receptor, muscarinic M2 receptor, and angiotensin 1–7 MAS receptor and induced distinct positive and negative chronotropic effects in the bioassay. Infected ferrets generated functional autoantibodies against β2-adrenoceptor and muscarinic M2 receptor and presented bioactivity similar to that in cattle. (4) Conclusions: This study uncovers functional autoantibodies targeting G-protein-coupled receptors in cattle and ferrets post-SARS-CoV-2 infection, with implications for cardiovascular function.

## 1. Introduction

Coronavirus disease 2019 (COVID-19), which is provoked by severe acute respiratory syndrome coronavirus 2 (SARS-CoV-2), has put a strain on healthcare systems globally. The initial symptoms of this infection include fever, dyspnea, cough, myalgia, and fatigue [1,2]. The virus targets the respiratory system and causes acute respiratory distress syndrome in patients with severe infections. Certain patients with SARS-CoV-2 infection have experienced long-lasting symptoms of COVID-19 that lasted for 2–3 months after the initial SARS-CoV-2 infection. The most frequently reported symptoms were chronic fatigue, dyspnea, chest and muscle pain, and neurological disorders that include brain fog, smell and taste dysfunction, and headache [3]. However, the mechanisms involved in the development of this distinct long-COVID and the numerous symptoms are unclear.

In recent years, there has been increasing evidence suggesting that autoimmune processes may play a role in the development of the various symptoms experienced by long-COVID patients. Studies have shown that SARS-CoV-2 patients who experienced long-COVID symptoms are characterized by the presence of autoantibodies (AAB) directed against extracellular antigens, immunomodulatory proteins such as cytokines and chemokines, and G-protein-coupled receptors (GPCR) [4,5,6]. Long-COVID patient sera displayed an AAB pattern that includes functional autoantibodies (fAAB) against β2-adrenoceptor, angiotensin II AT1 receptor, angiotensin 1–7 MAS receptor, and muscarinic M2 receptor [6]. Such antibody patterns have been detected in numerous long-COVID patients studied. Functional autoantibodies (fAAB) can activate their target receptors similarly to natural agonists, even though they bind to different receptor sites. This implies that fAAB can initiate receptor responses akin to normal physiological processes. The agonist-like fAAB identifies binding sites on the first or second extracellular loop of the receptors [7,8], while on the other hand, the classical agonists recognize an epitope localized in a pore formed by the seven membrane-spanning domains of the GPCR. However, in contrast to the classical agonists, the fAAB prevents the desensitization of the GPCR and provokes a permanent stimulation of the receptor-mediated signaled cascades. This permanent activation of the receptor causes a hormonal imbalance and may represent a pathogenic factor that could be responsible for the different symptoms seen in patients with long-COVID [9]. 

However, thus far, it has not been known whether the fAAB is produced as a consequence of the virus infection. To address this, we conducted a study on cattle and ferrets, two animal models known for their differing susceptibilities to SARS-CoV-2. Cattle show low susceptibility to SARS-CoV-2 following experimental infection [9,10], and ferrets can be readily infected with the wild-type virus and most of the variants of concern (VOCs) and transmit the virus to conspecifics [10,11,12,13,14,15].

The primary objective of our study was to ascertain whether experimental SARS-CoV-2 infection in cattle and ferrets leads to the formation of functional autoantibodies (fAAB). Understanding the interplay between viral infection and fAAB production in these animals may offer insights into similar phenomena in humans.

## 2. Materials and Methods

### 2.1. Serum Sample Preparation

We investigated the serum samples of nine cattle that were part of a previous experimental infection study that examined the susceptibility of cattle to SARS-CoV-2 infection and characterized the course of infection (Authorization no. MV/TSD/7221.3-2-010/18 of the State Office of Agriculture, Food Safety and Fisheries in Mecklenburg-Western Pomerania, Germany) [10]. Among these, six were intra-nasally inoculated with the SARS-CoV-2 strain 2019_nCoV Muc-IMB-1, and the remaining three cattle were kept as uninfected contact animals to investigate intraspecies virus transmission [10]. SARS-CoV-2 inoculation led to low-level viral replication in two out of six animals (animal numbers 776 and 768), followed by a specific seroconversion [10]. In our investigations, we used serum samples from this study. We used samples collected from all animals before SARS-CoV-2 inoculation and at 20 days post-infection (dpi). In addition, serum collected from three ferrets at 21 days post-infection (dpi) [12] and from three uninfected control ferrets were included.

The serum samples were heat-inactivated in a water bath at 56 °C for 1 h. Thereafter, the samples were subjected to dialysis with phosphate-buffered physiologic salt solution (pH 7.4) for 24 h to remove any small biologically active molecules. The serum samples were stored at −20 °C. 

### 2.2. Cell Preparation

Cardiomyocytes (CMs) used in the bioassay were prepared as described previously by Wallukat and Schimke [7] with the permission of Y9004/19, LAGESO, Berlin, Germany. Small sterile pieces of the ventricle from neonatal rat hearts were dissociated using crude trypsin at 37 °C. The isolated cells were treated with ice-cold calf serum and centrifuged. Subsequently, 2.4 million cells were seeded onto a culture flask of 12.5 cm^2^ and maintained in culture medium SM20-I (PAN Biotech, Aidenbach, Germany) supplemented with 10% neonatal calf serum (Serana GmbH, Pessin, Germany). After two days in culture, spontaneously beating cardiomyocytes were observed, and on day 4, the cells were ready for use in experiments. 

### 2.3. Bioassay for Measurement of Functional Autoantibodies

Initially, the beating rate of CMs was measured at baseline on a heated stage (37 °C) using an inverted microscope (Zeiss, Jena, Germany) at six different marked points of the culture flask. Serum or IgG preparations were then added to the CMs and incubated for 1 h. After incubation, the beating rate of the cells was reevaluated at the six previously marked locations. The difference between the basal beating rate and the beating rate after IgG incubation served as an indicator of the CM’s positive or negative response to the autoantibodies.

If the serum contained more than one fAAB with positive chronotropic and/or negative chronotropic activity, then the CMs were treated with antagonists to block the corresponding receptors. To measure the positive chronotropic response of the fAAB, the CMs were pretreated with 1 µM atropine (Sigma-Aldrich, St. Louis, MO, USA) and 1 µM A779 (Sigma-Aldrich) supplemented to block the muscarinic M2 receptor and the angiotensin 1–7 MAS receptor. In the case of the negative chronotropic response, the cells were pretreated with 0.1µM ICI118.551, 1 µM Losartan, 0.1 µM J113379, and 1 µM prazosin (Sigma-Aldrich, TOCRIS) supplemented to block the positive chronotropic action of the fAAB. As the serum could contain fAAB with both positive chronotropic and negative chronotropic activity, the receptors on the CMs were initially blocked by Atropine and A779 and ICI118.551, J113379, losartan, and prazosin, respectively.

### 2.4. Identification of the fAAB Binding Site on the Receptors

To identify the epitope of the AAB targeting the β2-adrenoceptor or the angiotensin 1–7 MAS receptor, we treated the fAAB with peptides that correspond to the first or second extracellular loop of both receptors, which are known as the binding sites of the fAAB on the extracellular domains of the GPCRs. It is important to mention that the homology of the extracellular domains of GPCRs between rats and other mammalian species is relatively high. Peptides corresponding to the first ^96^MKMWTFGNFWCEFWT^110^ and the second extracellular loop ^172^HWYRATHQEAINCYANETCCDFFTNQ^197^ of the β2-adrenoceptor were used (Biosynthan, Berlin, Germany). As for the human angiotensin 1–7 MAS receptor, the peptides representing its first and second extracellular loops are ^96^LSIDYALDYELSSGHYYI^113^ and ^173^DREEESHSRNDCRAVIIF^191^ (Biosynthan, Berlin, Germany), respectively. The peptide that effectively neutralized the AAB activity would represent the receptor peptide that was recognized by the fAAB.

### 2.5. Statistical Analyses

Statistical analyses were performed using the student-paired *t*-test. The significance threshold was set a priori at *p* < 0.05. All results are presented as mean ± standard error mean (SEM). The number of replicates and significant *p*-values are given in the figure legends. 

## 3. Results

**Figure 1:**
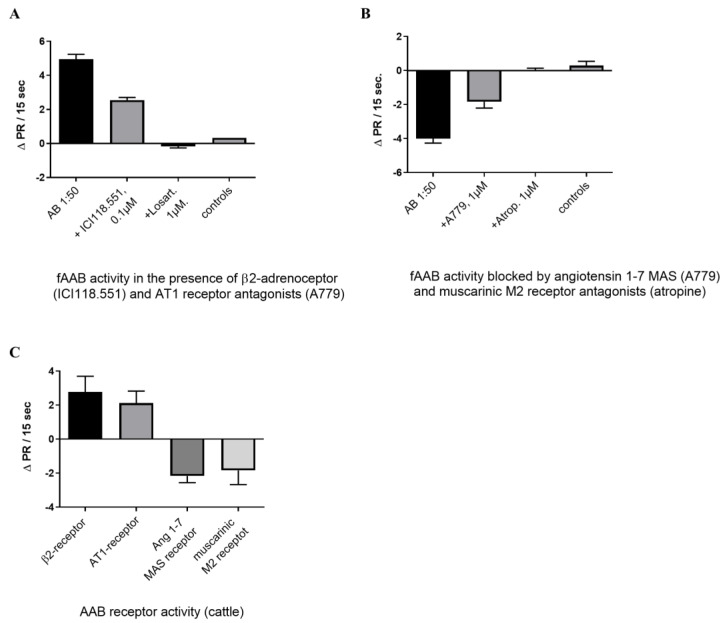
The serum of cattle with virus replication contains fAAB against the β2-adrenoceptor, the angiotensin AT1 receptor, the angiotensin1–7 MAS receptor, and the muscarinic M2 receptor. (**A**) Identification and inhibition of the fAAB activity by the antagonists of the β2-adrenoreceptor (ICI118.551) and the angiotensin II AT1-receptor (losartan) and compared to the controls. The cells were pretreated with the MAS receptor antagonist A779 and the muscarinic M2 receptor antagonist atropine to block the activity of the negative chronotropic AAB (*n* = 3, significance between column 1 and column 2, *p* < 0.002; between column 2 and column 4, *p* < 0.001). (**B**) Inhibition of the negative chronotropic AAB activity by A779 and atropine (*n* = 5, significance between column 1 and column 2 *p* < 0.002; between column 2 and column 4 *p* < 0.003). The cells were pretreated with ICI118.551, Losartan, Prazosin, and J113397 to block the positive chronotropic AAB activity. (**C**) Antibody pattern observed in sera of the infected cattle with virus replication.

Among the cattle inoculated with the virus, only two out of six showed viral replication [10] and tested positive for fAAB. Cattle that were infected with SARS-CoV-2 produced four distinct fAABs targeted at GPCRs. These fAABs specifically recognize the β2-adrenoceptor, angiotensin II AT1 receptor, muscarinic M2 receptor, and angiotensin 1–7 MAS receptor. Notably, the fAAB directed against the β2-adrenoceptor and angiotensin II AT1 receptor induced a positive chronotropic effect in the bioassay. The observed effect was partially inhibited by the β2-adrenoceptor inhibitor ICI118.551 and the antagonist of the angiotensin II AT1 receptor losartan (Figure 1A). ICI118.551 and losartan show that they only partially antagonize the effects induced by the fAAB. These findings suggest that two distinct fAABs are responsible for activating the cardiomyocytes. In contrast, the stimulation of cardiomyocytes with the fAABs targeting the muscarinic M2 and angiotensin 1–7 MAS receptor induces a negative chronotropic response, which could be blocked by A779, an angiotensin 1–7 MAS receptor antagonist, and by the blocker of the muscarinic M2 receptor atropine (Figure 1B). We could observe the fAAB pattern of the four different GPCR-fAAB in the sera (Figure 1C). 

**Figure 2:**
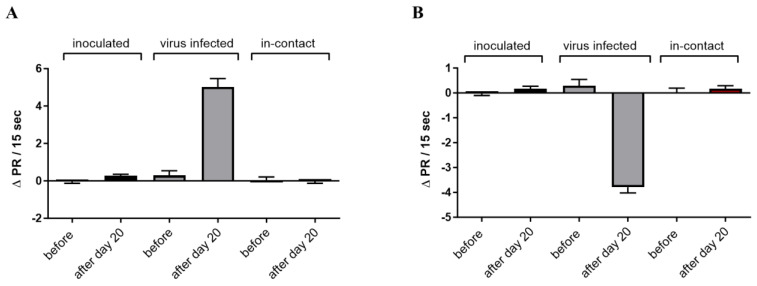
The effect of the immunoglobulins from cattle before and 20 days after virus inoculation. Only two out of six inoculated animals replicated the virus and generated fAAB against GPCR. The four inoculated cattle without virus replication and the three in-contact cattle did not develop fAAB against GPCR (*n* = 6 to 8). (**A**) The positive chronotropic fAAB activity is realized via the β2-adrenoceptor and the AT1 receptor, *p* < 0.001. (**B**) The negative chronotropic fAABs activate the angiotensin 1-7 MAS- and the muscarinic M2 receptor, *p* < 0.001.

All the cattle sera examined before the SARS-CoV-2 inoculation tested negative for fAAB (Table 1). The same applied to the other animals investigated on day 20, except for cattle 768 and 776. These two cattle displayed virus augmentation and the development of fAABs against GPCRs following the SARS-CoV-2 inoculation (Figure 2A,B). Figure 2A illustrates the combined impact of fAAB activity via the β2-adrenoceptor and the angiotensin II AT1 receptor, while Figure 2B displays the data associated with fAAB activity on the muscarinic M2 receptor and the angiotensin 1–7 MAS receptor. However, the three in-contact cattle tested negative for AABs both on days 0 and 20. 

**Figure 3:**
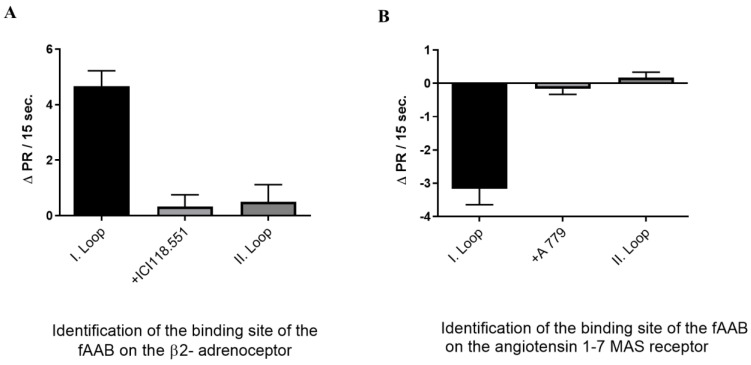
Identification of the binding site of cattle fAAB. (**A**) The fAABs recognize an epitope on the second extracellular domain of the β2-adrenoceptor (*n* = 6, significance between column 1 and column 2 and 3, *p* < 0.001), (**B**) and the angiotensin 1-7 MAS receptor (*n* = 6, significance between column 1 and column 2 and 3, *p* < 0.001). The fAABs were pretreated with peptides corresponding to the first or second extracellular loop of receptors of the β2-adrenoceptor and the angiotensin 1-7 MAS receptor.

The two AAB-positive cattle sera were used in the experiments conducted to identify the extracellular domain to which the fAABs bind. These experiments were performed for both the β2-adrenoceptor and the angiotensin 1–7 Mas receptor AAB. The fAABs were pretreated with peptides that corresponded to the second extracellular loop of these receptors (Figure 3A,B). Both fAABs were effectively neutralized by a peptide derived from the second extracellular loop. Consequently, we infer that the fAABs targeting both the β2-adrenoceptor and the angiotensin 1–7 Mas receptor likely recognize an epitope situated on the second extracellular loop of their respective receptors.

**Figure 4:**
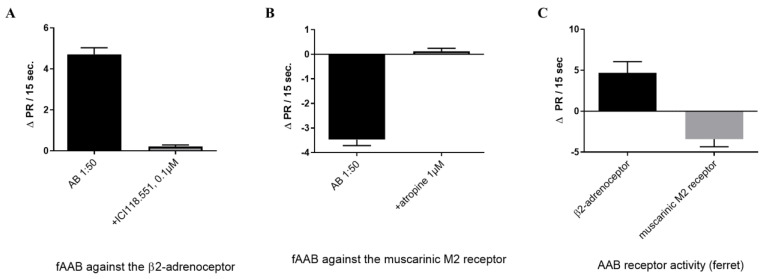
All SARS-CoV-2 infected ferrets generate fAAB against the β2-adrenoceptor and the muscarinic M2 receptor measured on day 21 after the infection. The fAAB activation was blocked by the (**A**) antagonist of the β2-adrenoceptor ICI118.551 (*n* = 11), *p* < 0.001, and (**B**) the inhibitor of the muscarinic M2 receptor atropine (*n* = 6), *p* < 0.001. (**C**) The antibody pattern of SARS-CoV-2 infected ferrets. The myocyte culture was pretreated as described before in Figure 1.

Unlike cattle, all ferrets infected with SARS-CoV-2 generated fAABs against GPCRs. In the sera of the three infected ferrets, fAABs targeting the β2-adrenoceptor and the muscarinic M2 receptor were detected (Figure 4A,B). As expected, the serum from the control ferrets did not exhibit any fAAB activity (Figure 3A, Table 2). On day 21 after inoculation, the positive chronotropic and negative chronotropic activities of these fAABs were measured and could be blocked by the corresponding receptor antagonists, ICI118.551, and atropine (Figure 4A,B and Table 2).

## 4. Discussion

Our research findings demonstrate that experimental inoculation of cattle and ferrets with SARS-CoV-2 can lead to the formation of fAAB activity targeted against GPCR. Among the experimentally inoculated cattle, only two out of six cattle generated fAAB against GPCR. In this study, it is essential to acknowledge and justify the relatively small sample size employed in our investigation. The rationale behind this limitation stems from the unique and challenging nature of our research objectives. We sought to explore the production and activity of functional autoantibodies targeting G-protein-coupled receptors following SARS-CoV-2 infection in selected animal species, specifically cattle and ferrets. Given the experimental nature of our study, it was imperative to work with animal models that closely mimic human responses to the virus. However, acquiring and maintaining such models for extensive experimentation can be logistically complex. It is important to note that our study utilized pre-existing samples from previous experimental infection investigations, and as a result, we were constrained by the group sizes inherent in those datasets. In the case of the cattle study, the determination of the number of animals to include in the research was based on a method utilizing a binomial probability distribution. The central aim behind this calculation was to maximize our ability to detect virulence. Despite these constraints, our study successfully identified and characterized functional autoantibodies in both cattle and ferrets post-SARS-CoV-2 infection, shedding light on their potential implications for cardiovascular function.

The formation of fAAB was observed only in cattle that exhibited virus replication, while no fAAB activity was observed in animals without virus augmentation. In our experiments, we added the prepared serum samples to the bioassay and identified the different fAABs by the addition of a specific antagonist, which blocked the functional activity of the AABs. The fAABs found in the two cattle sera recognize the β2-adrenoceptor, angiotensin II AT1 receptor, muscarinic M2 receptor, and angiotensin 1–7 MAS. Similar antibody patterns have been frequently observed in humans infected with SARS-CoV-2 who developed long-COVID symptoms [6,16,17]. The infected cattle that showed no virus reproduction and the three non-infected in-contact cattle were fAAB negative. Moreover, the experiments in which the binding site of the fAAB to the β2-adrenoceptor and the angiotensin 1–7 MAS receptor was analyzed indicate that the fAAB recognizes an epitope on the second extracellular loop of these receptors. 

In the SARS-CoV-2 infection experiments, it was observed that, unlike cattle, most ferrets were infected with SARS-CoV-2, irrespective of the used virus strain [15]. However, ferrets with a high virus replication rate developed only slight clinical signs. These animals showed mild rhinitis and SARS-CoV-2 reactive antibodies after 21 days [10]. In addition to the SARS-CoV-2 antibodies, the ferrets generated fAAB against GPCR. Two fAABs were detectable in the ferret serum and were specifically bound to the β2-adrenoceptor and the muscarinic M2 receptor. A similar antibody pattern was observed in human patients infected with SARS-CoV-2 who developed long-COVID-19 symptoms weeks/month after infection [6]. However, the exact time after the virus infection, when fAABs are formed in humans, remains unclear. It is indeed remarkable that animals infected experimentally with SARS-CoV-2 show the early development of fAABs targeting GPCR. In our study, we were able to demonstrate that serum samples from the ferrets on day 21 and two of the cattle on day 20 tested positive for fAABs following the experimental inoculation with SARS-CoV-2. These data were summarized in Table 1 and Table 2. Table 1 shows that only two cattle with virus augmentation develop fAABs against GPCR. The other cattle showed no fAAB activity on day 0 or day 20. This applied also to the in-contact cattle. Table 2 represents the data obtained for the ferrets. In the sera of the control ferrets, no fAAB activity was detectable. However, all infected animals investigated were fAAB positive on day 21 after inoculation.

The formation of functional autoantibodies (fAAB) activity targeted against G-protein-coupled receptors (GPCR) in cattle and ferrets following experimental inoculation with SARS-CoV-2 may be attributed to the complex interplay between the virus and the host’s immune system. There are a couple of potential explanations for this phenomenon. Firstly, SARS-CoV-2 might contain structural components or proteins that resemble the host’s GPCR receptors, triggering the generation of autoantibodies as a component of the host’s immune reaction. Secondly, the virus-induced inflammatory response could also contribute to the development of autoantibodies. The inflammatory mediators may interact with GPCRs, possibly resulting in the production of these autoantibodies. Further research is needed to pinpoint the exact mechanisms behind the development of fAAB against GPCR in SARS-CoV-2-infected animals.

Furthermore, our data clearly demonstrate that only cattle that show a virus reproduction generated fAAB against the identified GPCR. These findings suggest that the virus infection could be one of the contributing factors leading to the formation of fAABs. It is well-established that virus infections are usually associated with an amplified production of proinflammatory cytokines. Such cytokine storms are often observed in patients with severe SARS-CoV-2 infection [18,19,20,21,22,23,24]. In a study using a rat model of preeclampsia, reduced placental blood flow (RUPP-model) led to oxidative stress and increased proinflammatory cytokines like IL-6, IL-17, and TNFα. These cytokines appeared linked to the formation of fAABs targeting the angiotensin II AT1 receptor [20,23]. Treatment with a recombinant soluble IL-17 receptor eliminated fAAB formation [24]. Administering Th17 cells or IL-17 to normal pregnant rats led to fAAB development against the AT1 receptor, causing preeclampsia-like symptoms and mitochondrial dysfunction [20,24,25,26,27,28]. Some of these normal pregnant rats generate additional fAAB against the ETA receptor, an fAAB that was identified in women with severe preeclampsia and HELLP syndrome [29,30]. This antibody formation in the third trimester correlated with elevated IL-17 levels in the first trimester [31]. Thus, a potential link between the elevated proinflammatory cytokines and the generation of fAABs targeting GPCRs was hypothesized.

It should be noted that infecting cattle with SARS-CoV-2 proved to be challenging. Only two out of the six inoculated cattle showed virus production [10]. Additionally, SARS-CoV-2 was not transferred to the three non-infected in-contact cattle. These findings are in line with a study that reported an extremely low infection rate of cattle [32]. In contrast, the ferrets show a high infection rate, and it was observed that the virus was also transmitted to in-contact ferrets [10,15,32]. 

Investigations into the specific mechanisms underlying the generation of autoantibodies in response to SARS-CoV-2 infection could pave the way for identifying specific pathways or factors that could be targeted for intervention or prevention in the future. Therefore, it becomes crucial to gain a comprehensive understanding of how inflammatory cytokines regulate the immune response and contribute to the formation of autoantibodies against GPCR. Additionally, it is of paramount importance to investigate whether these agonist-like fAABs play a significant role in the development of the diverse symptoms observed in long-COVID patients. In this context, it is noteworthy that the application of the aptamer BC007, which demonstrated in vitro neutralization of fAAB in cattle and ferrets (data not presented here), resulted in the elimination of fAAB in a therapeutic experiment involving humans, as documented in a case report by Hohberger et al. [33]. This report linked the neutralization of fAAB by BC007 to a noticeable improvement in the symptoms of individuals dealing with long-COVID. Hence, there is a pressing need to explore and introduce novel therapeutic approaches for managing long-COVID and associated fatigue symptoms, given the potential significance of such interventions [34].

Furthermore, investigations into the role of these functional autoantibodies may provide insights into SARS-CoV-2-related cardiovascular complications and potential therapeutic targets for intervention.

## 5. Conclusions

Our research findings suggest that when cattle and ferrets are experimentally exposed to SARS-CoV-2, it can trigger the production of fAABs targeting GPCR. The fAAB formation was only seen in animals exhibiting a viral replication but not those without virus augmentation. The relatively early fAAB induction after the experimental virus infection and the formation of fAAB against vascular active receptors supports our idea that the fAAB against GPCR may play an important role in the development and persistence of the different symptoms observed in long-COVID and post-COVID-19 patients.

## Figures and Tables

**Table 1:** Antibody activity before and after the SARS-CoV-2 inoculation in cattle.

Cattle Number	Day 0	Day 20
Inoculated cattle		
776	0.17	4.69
768	0.59	5.32
766	−0.25	0.00
770	0.17	0.00
771	0.00	0.50
842	0.00	0.17
In−contact cattle		
774	−0.17	0.17
773	−0.33	0.42
777	0.42	−0.09

The total fAAB activity was realized via the β2-adrenoceptor and the angiotensin AT1 receptor.

**Table 2:** Antibody activity after the SARS-CoV-2 virus inoculation in ferrets.

Ferret	Day 0	Day 21
1	0.25	5.07
2	0.09	4.95
3	0.09	4.00

The total fAAB activity was realized via the β2-adrenoceptor.

## Data Availability

All data generated from this study are included in the manuscript.
